# Biofilm Associated Persistence and Drug Tolerance in Mycobacteria Within Host Microenvironments

**DOI:** 10.1111/apm.70166

**Published:** 2026-03-10

**Authors:** Lourdes Serrano Garcia, Cesar Augusto Roque‐Borda, Fernando Rogério Pavan, Marlus Chorilli

**Affiliations:** ^1^ School of Pharmaceutical Sciences São Paulo State University (UNESP) Araraquara Brazil; ^2^ Vicerrectorado de Investigación Universidad Católica de Santa María Arequipa Peru

**Keywords:** biofilm‐associated persistence, drug tolerance, granuloma biology, host microenvironment, *Mycobacterium tuberculosis*

## Abstract

Biofilms formed by mycobacteria, particularly *Mycobacterium tuberculosis* (Mtb), represent a major challenge in tuberculosis (TB) treatment due to their highly organized structure and their capacity to induce phenotypic drug tolerance. These three‐dimensional bacterial aggregates are embedded in a self‐produced extracellular matrix that restricts antibiotic penetration and shields bacilli from host immune responses. The resulting spatial and physiological heterogeneity within biofilms generates microenvironments that favor slow‐growing or non‐replicating cells, markedly reducing the efficacy of conventional antimicrobial therapies. Increasing experimental and clinical evidence supports the presence of biofilm‐like mycobacterial communities in TB lesions, linking this growth mode to disease chronicity, treatment failure, and relapse. This review aims to provide an integrated overview of the biological and physiological states adopted by mycobacteria within biofilm microenvironments, with particular emphasis on the mechanisms underlying biofilm‐associated drug tolerance. In addition, it critically discusses therapeutic strategies designed to overcome this tolerance, focusing on synergistic antibiotic combinations and peptide–antibiotic therapies that directly disrupt biofilm architecture, enhance drug penetration, or sensitize biofilm‐embedded bacilli to antimicrobial killing.

## 1 Introduction

Tuberculosis (TB), caused by *Mycobacterium tuberculosis* (Mtb), remains one of the most persistent infectious diseases worldwide, posing a major threat to global health despite decades of medical advances [1]. The emergence of multidrug‐resistant (MDR) and extensively drug‐resistant (XDR) strains has further complicated disease management, highlighting the limitations of conventional antibiotic therapies [2]. Recent studies have shown that Mtb, traditionally regarded as a free‐living intracellular pathogen, can also form structured multicellular communities embedded within a self‐produced extracellular matrix—referred to as biofilms [3]. This shift in the pathogen's lifestyle has significant implications for its physiology, promoting persistence, phenotypic tolerance, and evasion of immune responses [4, 5].

Biofilm formation has emerged as a key factor in the chronicity and recurrence of TB, as well as in the suboptimal response to standard chemotherapy [6]. Biofilm‐associated mycobacteria display reduced metabolic activity and altered gene expression, which hinder antibiotic penetration and efficacy. These characteristics underscore the need for a deeper understanding of the biological complexity of Mtb within host tissues [7]. Therefore, understanding the role of biofilm formation in the pathogenesis and persistence of TB is critical for improving disease management and for developing novel therapeutic strategies aimed at effective control and eradication of tuberculosis [8, 9, 10]. The lungs are a critical site for TB infection, where Mtb can establish biofilm‐like communities that protect the bacteria from host defenses and antibiotic penetration [11]. This biofilm‐associated state contributes to treatment failure and disease recurrence, as conventional therapies often fail to reach sufficient concentrations within these structured bacterial aggregates [12].

In response to these challenges, recent research has focused on several promising strategies to combat biofilm formation. This review provides an overview of the phenotypic and functional characteristics exhibited by mycobacteria in biofilm microenvironments, while also exploring three complementary therapeutic strategies with disruptive potential: antibiotic–antibiotic synergy, peptide–antibiotic combination therapies, and nanotechnology‐based drug delivery systems. These approaches not only enhance bactericidal activity but also target the structural and functional properties that underlie biofilm‐associated tolerance in Mtb.

## 2 Biofilm Foundations Overview

### 2.1 From Planktonic Cells to Polymicrobial Biofilms

Bacteria are commonly categorized as existing in two distinct life forms in nature: one as single, independent, free‐floating cells (planktonic), and the other as aggregates organized into microbial communities [13]. Biofilms are aggregates of bacteria encased in a protective matrix, which serves as a dynamic and functional network integrating both structural and regulatory components [14, 15]. The matrix encompasses the core extracellular matrix (ECM) proteins—such as collagen, laminins, fibronectin, elastin, and proteoglycans—as well as enzymes, growth factors, signaling molecules, and receptors involved in ECM remodeling, cell–matrix communication, and tissue homeostasis [16].

Historically, for a microbial structure to be classified as a biofilm, it had to be attached to biotic or abiotic surfaces, such as dental surfaces or medical devices, respectively [17]. However, non‐attached microbial aggregates are now also recognized as biofilms, particularly in clinical settings, where they are associated with respiratory tract infections due to impaired mucociliary clearance and persistent soft tissue infections [18]. Biofilms can consist of a single species or multiple, phylogenetically unrelated species [19]. The gradients within biofilm matrices generate micro‐niches, each colonized by microorganisms that have optimized their metabolism for the specific conditions [20]. Anaerobic microorganisms and cells more sensitive to environmental stressors, such as hazardous chemicals, pH, or physical damage, tend to be located in the deeper layers of the biofilm [19, 21].

Polymicrobial biofilms are defined as dense, surface‐attached communities in which different species of bacteria, fungi, and sometimes archaea coexist in close physical proximity, forming a shared extracellular matrix [22]. Within these polymicrobial biofilms, microbes engage in a range of interactions that shape community structure and function. Cooperative interactions, such as metabolic cross‐feeding and the sharing of “public goods” (e.g., extracellular polymeric substances, siderophores), can promote coexistence and spatial organization. Communication systems like quorum sensing coordinate collective behaviors, such as matrix production and virulence factor expression, across species [23].

At the same time, antagonistic interactions (e.g., nutrient competition, production of inhibitory molecules or bacteriocins, and enzymatic degradation of competitor signals) create spatial niches and can alter species dominance [24]. These interspecies interactions modulate clinically relevant traits, including spatial segregation or integration of partners in biofilms, changes in pathogen virulence or persistence, and alterations in antimicrobial susceptibility compared to single‐species biofilms [25]. It has been shown that biofilms, even when treated with antibiotics effective against planktonic bacteria or small bacterial communities at concentrations well above the Minimum Biofilm Inhibitory Concentration (MBIC)—which is defined as the lowest concentration of a drug needed to prevent bacterial growth—can remain unaffected in terms of structure. This allows continuous growth even after treatment [26].

### 2.2 Biofilm Formation Steps

Biofilm formation is a multistep process shaped by species‐specific traits, the physicochemical properties of the attachment surface, environmental conditions, and the regulated expression of biofilm‐associated genes [12]. The initial stage—cell adhesion—is typically reversible and begins when planktonic bacteria approach a surface through a combination of physicochemical interactions (e.g., van der Waals forces, electrostatic interactions, and hydrophobic effects). This weak attachment is subsequently reinforced by specific adhesins and surface structures, including pili/fimbriae and other adhesion proteins [27, 28]. Depending on the organism and the context, attachment may involve active mechanisms (motility and appendage‐mediated contact) as well as passive contributions from diffusion, sedimentation, and fluid flow [29]. Local environmental parameters, such as pH, temperature, ionic strength, and nutrient availability, strongly influence adhesion efficiency and early biofilm establishment [13].

Following attachment, adherent cells proliferate and form microcolonies, while producing extracellular polymeric substances (EPS; including polysaccharides, proteins, lipids, and extracellular DNA), which drive the transition to irreversible attachment and provide the scaffold for three‐dimensional architecture [30]. EPS deposition stabilizes cell–cell interactions, retains nutrients, and generates physicochemical gradients that promote functional differentiation within the developing community. At this stage, environmental cues and population density–dependent regulation converge, and quorum sensing (QS) frequently contributes to coordinated gene expression programs that support maturation and collective behavior [31, 32, 33].

QS relies on extracellular signaling molecules (autoinducers) that enable bacteria to sense local cell density and modulate gene expression accordingly, thereby influencing biofilm maturation and, in many species, dispersal [31, 33]. Additional mechanisms can facilitate long‐range communication and redox balancing within dense communities; for instance, conductive pili and other extracellular filaments (“nanowires”) have been described in some bacteria as structures that support electron transfer and may contribute to signal integration across gradients of nutrients, redox potential, and pH within biofilms [34]. The prevalence and functional relevance of such conductive networks, however, are species‐ and context‐dependent.

Finally, mature biofilms undergo detachment through active dispersal programs and/or physical erosion, releasing single cells or microcolonies that can seed new surfaces and contribute to dissemination on host tissues or medical devices [12, 35, 36]. This cyclical transition between sessile and planktonic states underlies the persistence of biofilm‐associated infections and contributes to recalcitrance to therapy, as the matrix and stratified physiology collectively limit antibiotic access and bactericidal activity [13].

### 2.3 Biofilm‐Related Impacts on Infectious Diseases

Accumulating evidence indicates that biofilm formation profoundly alters the course of infectious diseases by enabling bacteria to persist within host tissues and evade both immune surveillance and antimicrobial therapy. Notably, several pathogens traditionally classified as extracellular have been shown to adopt intracellular lifestyles in which they form organized microbial aggregates with biofilm‐like properties. Examples include uropathogenic *Escherichia coli, Pseudomonas aeruginosa, Borrelia burgdorferi, Moraxella catarrhalis*, nontypeable *Haemophilus influenzae, Streptococcus pneumoniae*, and group A *Streptococcus*, all of which can generate structured intracellular communities within host cells [37]. Such phenotypic plasticity complicates clinical diagnosis, contributes to recurrent infections, and often results in delayed or ineffective treatment [38].

Biofilm formation is a conserved strategy across both Gram‐positive and Gram‐negative bacteria and is frequently associated with reduced growth rates, metabolic heterogeneity, and stress‐adapted phenotypes that decrease susceptibility to antibiotics designed to target rapidly dividing cells [39]. Clinically relevant biofilm‐forming pathogens include *Staphylococcus aureus, Staphylococcus epidermidis, Enterococcus faecalis, viridans* group *streptococci, Escherichia coli*, *Proteus mirabilis, Klebsiella pneumoniae, Burkholderia cenocepacia, Clostridioides difficile, P. aeruginosa*, and *Vibrio cholerae* [40, 41, 42].

As a consequence, biofilms are implicated in a broad spectrum of chronic and recurrent infections, including native valve endocarditis, cystic fibrosis lung disease, periodontitis, otitis media, chronic rhinosinusitis, osteomyelitis, chronic bacterial prostatitis, non‐healing wounds, urinary tract and kidney infections, contact lens–associated keratitis, meningitis, and infections associated with implanted medical devices such as prosthetic joints, urinary catheters, and intravascular stents [43]. These infections typically persist despite apparently adequate antimicrobial therapy and intact host immune responses, reflecting the protective and adaptive properties of the biofilm state [44].

Several intrinsic features of biofilms contribute to immune evasion, infection relapse, tissue damage, and phenotypic tolerance to antimicrobials [45, 46, 47]. Reduced antibiotic efficacy arises from multiple, often coexisting mechanisms. The extracellular matrix acts as a physicochemical barrier that limits drug penetration and can sequester or repel antimicrobial molecules depending on its composition [48, 49]. In addition, biofilm‐associated cells may express antibiotic‐degrading enzymes, alter target availability, or adopt physiological states with reduced metabolic activity that render bactericidal agents less effective. Structural and phenotypic remodeling of bacterial cells within biofilms further reinforces tolerance and persistence [50].

At the regulatory level, biofilm formation and maintenance are controlled by interconnected genetic networks, including quorum‐sensing systems, cyclic dinucleotide signaling pathways (e.g., c‐di‐GMP), and small non‐coding RNAs. Among these, quorum sensing has emerged as a particularly attractive therapeutic target due to its central role in coordinating collective behaviors without directly affecting bacterial viability [51].

Despite the growing recognition of biofilms in human disease, no standardized diagnostic protocols exist for routine clinical detection [52]. Biofilm‐associated infections often remain subclinical for extended periods and are frequently identified only after systemic dissemination or bacteremia has occurred [53]. Consequently, the development and harmonization of experimental model systems across the biofilm research community are critical to improve reproducibility, facilitate translational studies, and support the rational design of antibiofilm therapeutic strategies [54] (Table 1).

**TABLE 1:** Models designed to investigate the formation, structure, and behavior of microbial biofilms.

Model type	Characteristics	Objectives	Model Examples	References
*In vitro* – Static	Microorganisms grow adhered to surfaces without flow. Static liquid medium; use of Petri dishes, microplates, and glass slides.	Study adhesion, biofilm formation, architecture, and growth kinetics.	*Acinetobacter baumannii* biofilm under static conditions; complex root caries biofilm on bovine enamel/rhizome with sucrose cycling.	[55]
*In vitro*—Dynamic	Systems with controlled medium flow over the biofilm surface, including shear forces, real‐time imaging, and microfluidics.	Analyze the effect of flow, shear forces, nutrient availability, and spatial organization of the biofilm.	*Pseudomonas aeruginosa* biofilm in flow systems on stainless steel; *Pseudomonas putida* biofilm in microfluidic channels under variable flow.	[56, 57]
Microcosms/Organoids	Adapted models to simulate more physiological environments: use of natural substrates, differentiated cells, and organoid structures.	Represent more realistic biological conditions, allow polymicrobial biofilms, and simulate human microenvironments.	Polymicrobial oral biofilm on natural scaffolds with enamel and saliva; wound biofilm in pig tissue; lung or intestinal human organoids.	[58, 59]
*In vivo*	Biofilm growth in living organisms (invertebrates or mammals), real host environment, and host–microorganism interactions.	Study host–microbe interactions, therapeutic efficacy, and clinically relevant models.	Biomaterial implant with *Staphylococcus aureus* in mice; *P. aeruginosa* lung biofilm in mice showing in vivo antimicrobial resistance evolution; otitis media in chinchilla; chronic wounds in pig; osteomyelitis in mice.	[60, 61]
*In silico*	Computational/mathematical models simulating biofilm growth, structure, and dynamics (nutrients, diffusion, cell interactions).	Predict biofilm development, optimize control strategies, and explore hypothetical scenarios at low cost.	Quorum sensing signaling *in silico* models; reaction–diffusion models; Monod growth models; hybrid multi‐species biofilm models in bioreactors.	[62, 63]

### 2.4 Cross‐Kingdom Interactions in Bacterial Biofilms

Beyond mono‐ and polymicrobial bacterial biofilms, bacteria frequently participate in complex cross‐kingdom communities with fungi, forming structured multicellular aggregates embedded within a shared extracellular matrix [64]. These mixed bacterial–fungal biofilms display emergent properties that differ markedly from those of single‐species communities, including altered architecture, metabolic cooperation, and increased tolerance to antimicrobial and antifungal agents [65].

Cross‐kingdom interactions within biofilms are dynamic and give rise to intricate ecological relationships that contribute to the structural and functional complexity of microbial consortia [66]. In many settings, filamentous fungal elements provide a physical scaffold that facilitates bacterial adhesion, spatial organization, and biofilm stability. Conversely, competitive interactions also occur, whereby fungi inhibit bacterial biofilm development through nutrient competition or the secretion of antimicrobial secondary metabolites [67, 68].

Several clinically relevant bacterial species are known to form mixed biofilms with fungi, often resulting in enhanced antimicrobial tolerance or altered pathogenic behavior [69]. The interaction between *Staphylococcus aureus* and *Candida albicans* is among the best‐characterized examples, in which co‐culture leads to increased bacterial tolerance to antibiotics such as vancomycin [70]. Similarly, mixed biofilms formed by *S. aureus* or *Staphylococcus epidermidis* with filamentous fungi, including Fusarium solani, exhibit increased resistance when established on human corneal tissues [71]. Interactions between *P. aeruginosa, Stenotrophomonas maltophilia*, and *Aspergillus fumigatus* further illustrate the context‐dependent nature of cross‐kingdom biofilms, which can involve antagonism, metabolic competition, or structural cooperation in matrix production and biofilm organization [72].

### 2.5 Host–Pathogen Interplay

In addition to their intrinsic tolerance to antimicrobial agents, biofilms display a remarkable capacity to withstand host immune defenses. The biofilm matrix functions as a physical and biochemical barrier that restricts immune cell infiltration, limits antibody and complement access, and protects embedded microorganisms from phagocytosis and oxidative killing [73]. These properties enable pathogens within biofilms to persist in host tissues despite sustained immune activation.

Historically, most studies of host immune responses to bacterial infection have focused on planktonic organisms. However, accumulating in vivo and in vitro evidence indicates that biofilms elicit distinct innate and adaptive immune responses compared to free‐living bacteria [74]. Biofilm‐associated infections are often characterized by chronic inflammation, ineffective microbial clearance, and tissue damage, reflecting a state of immune engagement without resolution.

Recent studies have begun to elucidate the mechanisms by which immune cells recognize and respond to biofilm‐associated pathogens, including altered pattern‐recognition receptor signaling, impaired neutrophil function, and skewed macrophage activation states (Table 2). Understanding these host–biofilm interactions is essential for identifying immunomodulatory strategies that complement antimicrobial or antibiofilm therapies and improve infection outcomes.

**TABLE 2:** Immune defense responses triggered by biofilms from different microbial species.

Microbial species	Models	Innate response	Adaptative response	References
*P. aeruginosa*	Murine keratitis model; human neutrophils ex vivo	Neutrophil extracellular traps (NETs) formation; ROS production; cytokine release (IL‐8, TNF‐α).	Limited adaptive activation due to immune cell sequestration in biofilm matrix.	[75]
*P. aeruginosa* (QS mutants)	Human neutrophils in vitro; mouse infection model	Altered NETosis and degranulation depending on quorum‐sensing phenotype.	Impaired Th17 responses and antibody recognition.	[76]
*Staphylococcus aureus*	Macrophages (murine & human) in vitro	Biofilm‐conditioned medium suppresses macrophage activation and cytokine expression (IL‐1β, TNF‐α).	Reduced antigen presentation and T‐cell stimulation.	[77]
*Staphylococcus aureus* (SaeRS‐regulated biofilm)	Macrophage co‐cultures; murine infection model	Inhibition of NF‐κB and TLR2 signaling in macrophages and reduced ROS.	Diminished Th1/Th17 cytokines; immune tolerance.	[78]
*Staphylococcus epidermidis*	Human and murine macrophages; implant models	eDNA triggers TLR9; suppresses phagocytosis and induces immunometabolic tolerance.	Weak adaptive priming; decreased antibody titers in implant‐associated infection.	[79]
*Klebsiella pneumoniae*	Murine macrophages; clinical biofilm isolates	Reduced phagocytosis and ROS; altered TNF‐α, IL‐6 profiles.	Poor activation of T‐cell responses; chronic persistence.	[80]
*Uropathogenic Escherichia coli*	Murine UTI model; bladder epithelial cells	Intracellular biofilm‐like communities evade neutrophil recruitment; suppress IL‐1β and IL‐6.	Limited adaptive memory; recurrent infection linked to poor B‐cell activation.	[81]
*Candida albicans*	Human neutrophils in vitro; murine infection model	Fungal biofilm eRNA induces NETosis; ECM components modulate TLR2/4 activation.	Weak Th1 and Th17 activation; tolerance to fungal antigens.	[82]
*Candida auris*	Human neutrophils and macrophages; ex vivo and murine models	Strong inhibition of NET formation and phagocytosis; reduced ROS.	Low antibody recognition; impaired Th17‐mediated clearance.	[83]
*Porphyromonas gingivalis*	Human gingival epithelial cells; mouse periodontal model	Biofilm suppresses IL‐8 and defensin secretion; alters macrophage polarization.	Subversion of B‐cell and T‐cell responses; chronic low‐grade inflammation.	[84]
*Streptococcus mutans*	Human and murine macrophages; rat dental biofilm model	Outer membrane vesicles inhibit inflammasome activation and glycolytic reprogramming.	Weak IgA response; limited T‐cell activation in mucosal tissue.	[85]
*Mycobacterium tuberculosis*	In vitro pellicle biofilms; murine and primate granuloma models	Biofilm cells resist complement activation and macrophage killing.	Suppressed antigen presentation; delayed T‐cell response in granulomas.	[86]

Planktonic bacteria are detected by the innate immune system through pathogen recognition receptors (PRRs) that recognize pathogen‐associated molecular patterns (PAMPs), such as flagellin and lipopolysaccharide (LPS), which are sensed by Toll‐like receptor 5 and Toll‐like receptor 4, respectively. Biofilm‐associated bacteria can engage the same recognition pathways; however, their detection is substantially altered by the presence of an extracellular polymeric matrix that masks classical PAMPs and limits their accessibility to immune receptors [87]. In addition, biofilm growth is frequently associated with the downregulation or structural modification of PAMP expression, further reducing immune recognition [88]. Biofilm‐associated infections typically induce a sustained inflammatory state that involves both innate and adaptive immune responses due to their chronic nature. Despite ongoing immune activation, neither arm of immunity is able to completely eradicate biofilms, resulting instead in persistent inflammation, ineffective microbial clearance, and progressive collateral tissue damage.

Biofilm‐forming bacteria deploy multiple, overlapping strategies to evade host immune defenses and ensure long‐term persistence [89]. The extracellular polymeric matrix—composed primarily of polysaccharides, proteins, and extracellular DNA—acts as both a physical and chemical barrier that restricts the penetration of antibodies, complement components, and antimicrobial peptides [90, 91]. Within this protected microenvironment, bacteria often adopt slow‐growing or dormant physiological states, further reducing susceptibility to immune‐mediated killing [92]. In parallel, many species secrete immunomodulatory factors that suppress pro‐inflammatory cytokines such as tumor necrosis factor‐α (TNF‐α) and interleukin‐1β (IL‐1β), while promoting anti‐inflammatory signaling pathways, including interleukin‐10 (IL‐10) induction, thereby establishing localized immunosuppression [93]. Additional mechanisms, such as quorum‐sensing–mediated regulation, outer membrane vesicle release, surface antigen variation, and capsule formation, interfere with neutrophil activation, T‐cell proliferation, antibody binding, opsonization, and phagocytosis [35, 94].

Given the global burden of chronic biofilm‐associated infections, it is essential to examine how biofilm biology contributes to specific diseases of major clinical relevance. Among these, tuberculosis (TB) represents a paradigmatic case in which Mtb exhibits biofilm‐like behaviors that promote drug tolerance, immune evasion, and disease persistence. The following section therefore focuses on current models and evidence supporting biofilm formation in TB, and discusses the implications for pathogenesis and therapeutic intervention.

## 3 Biofilm in Mycobacteria

Mtb is a slow‐growing, aerobic, non‐motile, non–spore‐forming bacterium belonging to the phylum *Actinomycetota*, class *Actinomycetia*, order *Mycobacteriales*, and family *Mycobacteriaceae* [95, 96]. It is characterized as an acid‐fast bacillus due to the high lipid content of its cell wall, particularly mycolic acids, which confer resistance to decolorization by acid–alcohol following Ziehl–Neelsen staining [97]. The microorganism has a high G + C content genome and reproduces by binary fission. Unlike typical Gram‐positive bacteria, *M tuberculosis* possesses a complex, waxy envelope that limits nutrient uptake and contributes to its intracellular survival within host macrophages [98].

The genus *Mycobacterium* is a highly diverse group consisting of numerous rod‐shaped, acid‐fast, Gram‐positive bacteria [99]. The *Mycobacteriaceae* family includes more than 170 recognized species, comprising strict pathogens causing lethal diseases such as tuberculosis, opportunistic pathogens, and species that are non‐pathogenic depending on their ability to cause infection [100]. Mycobacteria are commonly classified as TB‐causing species (e.g., Mtb) and non‐tuberculous mycobacteria (NTM) based on pathogenesis, and alternatively as rapidly growing mycobacteria (RGM) or slowly growing mycobacteria based on their growth rate [101].

NTM are ubiquitous and are found to inhabit a diverse range of environments including water bodies like natural waters, drinking water distribution systems, and soils. Many NTM are major opportunistic pathogens such as *Mycobacterium abscessus* [102]. and *Mycobacterium avium* [103]. *M. abscessus* being a rapidly growing NTM, commonly causes chronic pulmonary disease in patients with underlying lung conditions. Among all the members of the genus Mycobacterium, *M tuberculosis* is a global healthcare concern as it is highly pathogenic and causes TB [4]. Despite the availability of anti‐tubercular drugs, TB remains one of the major causes of death among other infectious diseases. The ability of mycobacteria to form biofilms presents a formidable obstacle to effective treatment and diagnosis [104].

The transition of mycobacteria from a solitary, free‐floating (planktonic) existence to a structured, sessile community is a complex adaptive response to environmental pressures, triggered by a variety of cues encountered within the host [105]. This adaptive strategy allows the bacteria to cease their energy‐intensive planktonic growth and adopt a defensive, aggregated‐based lifestyle that is better suited for long‐term survival and persistence in the hostile host environment [106, 107]. For many years, our understanding of TB pathogenesis and drug efficacy was based on studies of Mtb in its disaggregated state. However, a paradigm shift is underway, recognizing that in its natural host environment, Mtb, like many other bacteria, predominantly exists within biofilms [108]. Beyond aggregation itself, biofilm‐associated persistence in mycobacteria is tightly coupled to adaptive remodeling of the cell envelope. Recent work has shown that the Rv0077c–Rv0078 regulatory module integrates environmental cues to reshape mycolic acid composition, partly through modulation of the Mce1 lipid‐uptake system. Derepression of Rv0077c alters the distribution of long‐chain mycolic acids and reorganizes the outer membrane architecture, generating phenotypes associated with altered permeability and shifts in antibiotic susceptibility. These findings highlight that biofilm‐associated tolerance in mycobacteria is not solely a physical consequence of extracellular matrix formation, but also emerges from active metabolic and structural reprogramming of the mycomembrane. Incorporating such regulatory and lipidomic adaptations into biofilm models is therefore essential for interpreting drug‐efficacy studies and for distinguishing true biofilm‐specific tolerance from envelope‐mediated phenotypic resistance [109].

### 3.1 In Vitro Biofilm Models to Study Mycobacteria Biofilms

The experimental context in TB research is characterized by a continued reliance on established in vitro and in vivo models [6], with an increasing integration of computational approaches [110] and rigorous systematic review methodologies [7] to synthesize findings from diverse studies. The choice of model system is dictated by a specific research question, ranging from basic cellular mechanisms to complex host interactions and clinical achievements [111]. Phenotypic drug tolerance in TB arises from mechanisms that allow a subset of bacterial cells to survive antibiotic treatment despite being genetically susceptible [112]. Two key contributors are the non‐replicating persistent (NRP) state, in which metabolically quiescent cells maintain minimal energy to persist under stress, and biofilm formation.

#### 3.1.1 Pellicle Biofilm Formation at the Liquid–Air Interface

Pellicle biofilms of Mtb develop through a series of well‐defined stages, during which the ECM acquires a lipid‐rich composition dominated by keto‐mycolic acids. These lipids contribute to the structural cohesion of the pellicle and are increasingly recognized as determinants of antibiotic tolerance. Pellicle biofilms enable longitudinal observation of biofilm maturation and have been used to evaluate the activity of candidate antimicrobials across distinct developmental stages. Their slow formation parallels the protracted course of disease and the emergence of persistent bacterial subpopulations in vivo, making this model relevant for studying chronic infection and drug tolerance [112].

Under in vitro conditions, Mtb forms pellicles at the liquid–air interface over approximately 5–7 weeks, progressing from initial surface adherence to the establishment of a stratified, highly hydrophobic biofilm. The mechanical integrity of these structures depends on the enrichment of keto‐mycolic acids within the ECM, which reduces permeability and enhances resilience to external stressors, including antibiotics [113]. Pellicles also accumulate phenotypically tolerant cells, providing a tractable system for dissecting persistence mechanisms. For example, the small molecule TCA1 retains activity against pellicle‐associated bacilli, including subpopulations that exhibit reduced metabolic activity, supporting its potential for targeting both replicating and non‐replicating Mtb s [114]. Subsequent studies have confirmed that pellicles contain a higher proportion of drug‐tolerant persisters than planktonic cultures, reinforcing the value of this model for screening compounds that target phenotypic tolerance [115].

More recent work has refined our understanding of the regulatory circuits governing pellicle development. Keto‐mycolic acids not only contribute to the physical properties of the ECM but also modulate host–pathogen interactions, underscoring their dual structural and immunomodulatory roles [116]. Regulatory proteins such as Lsr2, along with second messengers including c‐di‐GMP, orchestrate mycolic acid biosynthesis and biofilm maturation, linking global transcriptional control to the architecture and persistence of pellicles. Despite these advances, the pellicle model has limitations: biofilm formation is slow, heterogeneity across strains remains underexplored, and the extent to which in vitro pellicles recapitulate ECM organization in vivo is not fully resolved. Nonetheless, pellicle biofilms remain a physiologically relevant system for probing long‐term persistence, monitoring ECM remodeling, and evaluating therapeutics with activity against both actively replicating and drug‐tolerant Mtb populations [115, 116].

**FIGURE 1:**
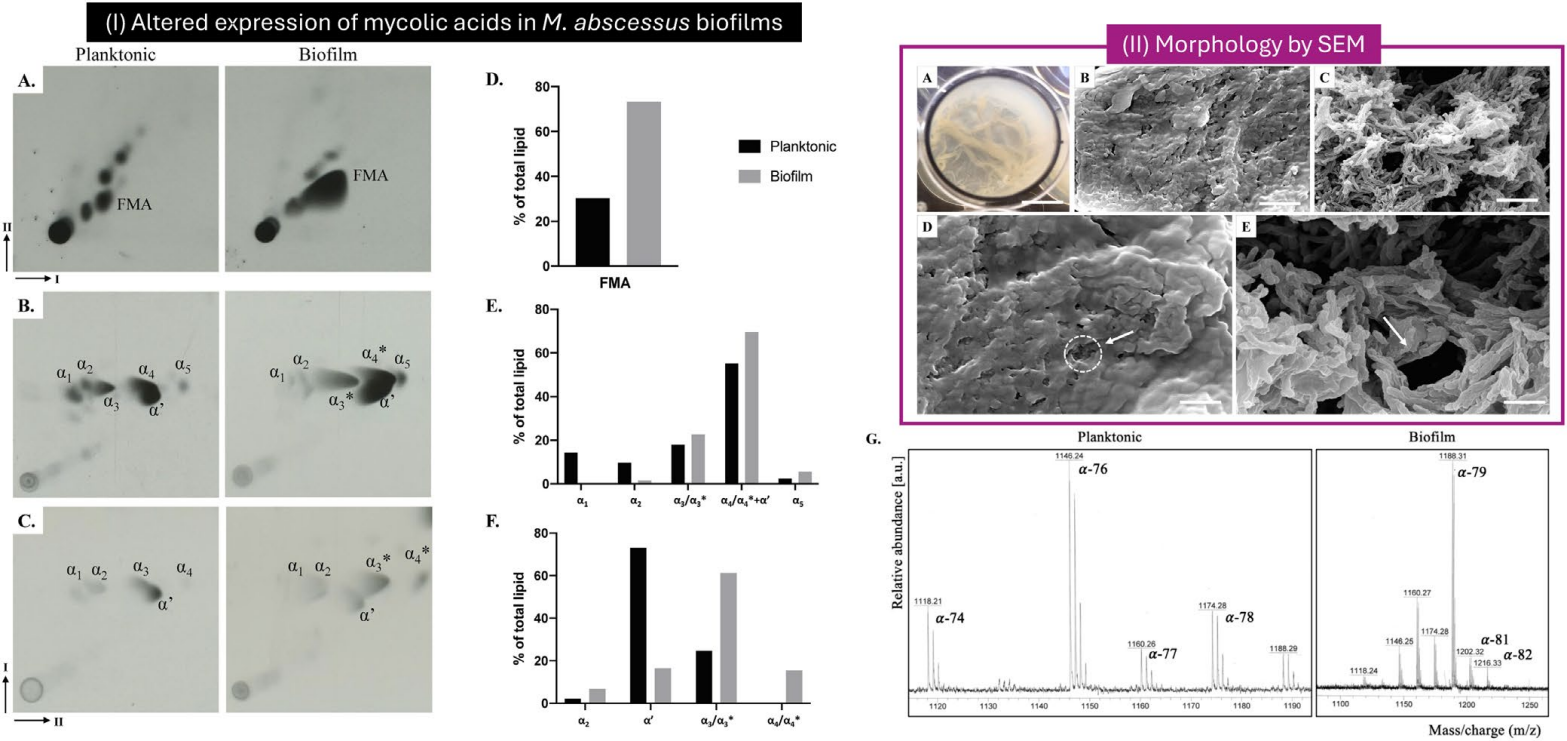
Structural and lipidomic differences between planktonic and biofilm‐associated *Mycobacterium abscessus* . (I) Altered expression of mycolic acids in biofilms. Representative 2D‐TLC analyses of total mycolic acids extracted from planktonic (left) and biofilm‐grown (right) cells (A–C). Free mycolic acids (FMA) increase substantially in biofilms (A), accompanied by redistribution of α‐mycolic acid subclasses (B) and shifts in α'‐mycolates (C). Quantification of lipid species (D–F) shows a marked enrichment of FMA and α_3_/α_3_* subclasses in biofilm conditions, alongside decreased relative abundance of α₁, α₂, and α₅ species, indicating biofilm‐specific remodeling of the mycolic acid pool. Mass spectrometry (G) further confirms elongation of α‐mycolates in biofilms, with prominent shifts from α‐74/76/77/78 in planktonic cells to longer‐chain α‐79/81/82 species in biofilms. (II) Pellicle morphology by scanning electron microscopy. Macroscopic and ultrastructural features of *M. abscessus* pellicles (A–E) reveal a highly porous, filamentous matrix enriched in extracellular material, contrasting with the smoother surface typical of many Mtb pellicles. SEM images highlight extensive folding, ECM accumulation, and voids characteristic of mature *M. abscessus* biofilms, with arrows indicating regions of dense extracellular material and structural heterogeneity. These features reflect species‐specific biofilm architecture and correlate with the lipid remodeling patterns shown in panel I. Figure adapted from Dokic et al. [117], The Cell Surface published under the Creative Commons CC‐BY license, which permits reuse, distribution, and adaptation provided the original work is properly cited.

Although pellicle formation has been most extensively characterized in Mtb, important insights into ECM architecture have also emerged from studies of nontuberculous mycobacteria such as *M. abscessus*. In *M. abscessus* [117], high‐resolution SEM imaging reveals a markedly porous and filamentous pellicle surface with dense ECM accumulation (Figure 1‐I). Confocal analyses further show that lipids dominate the ECM, accompanied by irregular and sparsely distributed proteins, carbohydrates, and extracellular DNA. While this general lipid‐rich ECM pattern is consistent with what has been described for Mtb, the surface architecture captured by SEM suggests a more porous and filamentous matrix in *M. abscessus* pellicles, whereas Mtb pellicles tend to form a denser and more compact surface layer dominated by keto‐mycolic acids. Beyond these morphological distinctions, *M. abscessus* exhibits biofilm‐specific remodeling of mycolic acid subclasses, including shifts in α‐MAME profiles and increases in free mycolic acids (Figure 1‐II), whereas Mtb predominantly regulates keto‐mycolic acid abundance to maintain pellicle stability. Together, these observations indicate that although ECM composition in mycobacterial biofilms shares conserved lipid‐rich features, the structural organization and lipid remodeling strategies differ across species. Such differences underscore the importance of species‐specific biofilm biology and caution against direct extrapolation of ECM properties from nontuberculous mycobacteria to Mtb.

#### 3.1.2 Leukocyte Lysate‐Induced Biofilms

Leukocyte lysate–induced biofilms form on surfaces when bacteria are exposed to culture media supplemented with leukocyte lysates, a condition designed to simulate the biochemical environment of caseous necrotic granulomas. Under these conditions, Mtb develops surface‐attached, matrix‐embedded communities that display pronounced tolerance to first‐line anti‐tuberculosis drugs. Importantly, these biofilms can be dispersed by treatment with DNase I or non‐ionic detergents such as Tween 80, demonstrating that the structural integrity of the extracellular matrix is a central determinant of phenotypic drug tolerance rather than stable genetic resistance [112]. The utility of this method lies in its ability to generate surface‐attached Mtb communities that closely resemble drug‐tolerant bacterial populations found within necrotic granulomas, thereby providing a biologically relevant platform to study antibiotic tolerance and to screen potential adjunctive therapies [112]. In a seminal experimental study, Mtb cultured under leukocyte lysate–supplemented conditions exhibited markedly increased tolerance to first‐line anti‐TB drugs, while enzymatic disruption of extracellular DNA or addition of Tween 80 led to biofilm dispersal and restoration of drug susceptibility, underscoring the critical role of matrix integrity and attachment in tolerance expression [118].

This model has also been exploited as a screening platform for antibiofilm compounds. In a complementary study, 2‐aminoimidazole (2‐AI) small molecules were shown to reverse phenotypic drug tolerance and restore susceptibility of biofilm‐associated Mtb to isoniazid in a dose‐dependent manner, highlighting the potential of matrix‐targeting agents as adjunct therapies capable of enhancing the efficacy of conventional anti‐tuberculosis regimens [119]. Although few original experimental studies in the past two years have directly applied leukocyte lysate–induced biofilm models to *M tuberculosis*, recent work in closely related non‐tuberculous mycobacteria supports the continued relevance of host component–driven biofilm systems. Notably, studies in *Mycobacterium avium* have demonstrated that exposure to host‐derived factors promotes the formation of surface‐attached, matrix‐embedded communities with enhanced antibiotic tolerance, reinforcing the concept that host‐associated microenvironments can drive biofilm‐mediated persistence in mycobacteria [120]. In parallel, recent analyses of tuberculosis experimental model systems emphasize the growing importance of host‐mimicking in vitro platforms—including immune component–supplemented cultures and granuloma‐associated environments—to investigate phenotypic drug tolerance and persistence mechanisms not captured by planktonic assays [121].

Collectively, while contemporary experimental evidence is currently stronger for non‐tuberculous mycobacteria, these findings support leukocyte lysate–induced biofilms as a promising translational model for Mtb. Although this approach was originally established and experimentally validated in Mtb, its direct application in TB research has been limited in recent years. Nevertheless, host component–driven biofilm models remain actively employed in other bacterial pathogens and in non‐tuberculous mycobacteria, where exposure to leukocyte‐derived material reproducibly promotes surface‐attached, matrix‐embedded communities with enhanced antibiotic tolerance. Recent experimental and conceptual advances in host‐mimicking biofilm systems therefore reinforce the biological relevance of leukocyte lysate–based approaches and highlight an underexplored opportunity to revisit and refine this model for Mtb under contemporary experimental settings.

#### 3.1.3 Thiol Reductive Stress‐Induced Biofilms

Thiol reductive stress (TRS)–induced biofilms form rapidly, typically within 29–30 h, when mycobacteria are exposed to reducing conditions. Depending on the culture setup, TRS can induce either submerged biofilms or pellicle‐like structures at the liquid–air interface. These biofilms are strongly attached to the substratum, resistant to mechanical disruption, and suitable for high‐resolution analyses of attachment, differentiation, and extracellular polymeric substance (EPS) synthesis using microscopy‐ and omics‐based approaches. Owing to their short formation time, TRS‐induced biofilms provide a practical experimental platform for drug screening compared with slower‐forming pellicle or leukocyte lysate–induced biofilms [112]. The TRS model was originally described by Trivedi et al. [122], who demonstrated that exposure of Mtb to 6 mM dithiothreitol triggers intracellular accumulation of free thiols and induces the formation of substratum‐attached biofilms enriched in cellulose. These biofilms develop within ~29 h and consist of metabolically active yet antibiotic‐tolerant bacteria. Biofilm formation under TRS conditions required active DNA, RNA, and protein synthesis and was associated with a limited but specific transcriptional response, indicating that this phenotype arises from a regulated physiological program rather than from nonspecific stress‐induced aggregation.

Subsequent work further refined this model by showing that media components such as bovine serum albumin enhance biofilm formation under reductive stress by acting as nucleation sites for extracellular matrix assembly, including polysaccharides and proteins, in both Mtb and *M smegmatis* [123]. Related observations in *M. bovis* BCG demonstrated that reductive stress induced by glutathione promotes bacterial persistence and tolerance to killing, supporting a broader role for thiol‐based redox pathways in mycobacterial survival [124].

Recent studies have reinforced the concept that biofilm growth in mycobacteria is not merely a structural state but a selectable and regulated phenotype. Experimental evolution coupled with transcriptome profiling in Mtb has revealed reproducible transcriptional rewiring during adaptation to biofilm growth, supporting the idea that biofilm‐associated tolerance can emerge from defined regulatory trajectories rather than from passive matrix shielding alone [109].

In parallel, although TRS‐triggered biofilms are still rarely applied directly to Mtb in contemporary experimental pipelines, redox‐ and host factor–driven biofilm paradigms remain active in non‐tuberculous mycobacteria. These studies demonstrate that exposure to host‐ or redox‐associated cues promotes stable, surface‐attached biofilm communities with enhanced tolerance, providing a pragmatic route to refine hypotheses and experimental readouts that can later be ported to TB‐relevant settings [125]. Mechanistically, emerging regulatory frameworks linking second messengers and global regulators to mycobacterial biofilm programs further justify revisiting TRS systems as fast, controllable platforms to interrogate matrix assembly and tolerance phenotypes under defined redox constraints [126, 127]. Finally, while pellicle, leukocyte lysate–induced, and TRS‐induced biofilms capture distinct aspects of extracellular persistence, none of these models incorporates a living airway surface. This limitation has motivated the development of epithelial‐associated biofilm systems under air–liquid interface conditions, which provide a more physiologically relevant framework to study early events of pulmonary colonization.

#### 3.1.4 Epithelial‐Associated Biofilms Under Air–Liquid Interface

Host‐associated biofilms represent a physiologically meaningful phenotype of Mtb that differs fundamentally from pellicle biofilms, leukocyte lysate‐induced communities, and TRS models. Recent work using differentiated primary bronchial epithelial cells (PBEC) grown at the air–liquid interface (ALI) has demonstrated that Mtb and related mycobacterial species can rapidly form structured, polysaccharide‐rich biofilms directly on airway surfaces [128]. In this system, bacteria encounter a stratified epithelium with mucus production, tight junctions, and an apical air‐exposed interface—conditions that reproduce early events of airway colonization far more accurately than classical in vitro platforms.

Confocal microscopy shows that initial adhesion occurs within the first 4–12 h, followed by coordinated expansion into multicellular aggregates by 24 h and the emergence of dense, spatially organized biofilm structures by day 7. As illustrated in Figure 2 of Barclay et al., scanning electron microscopy reveals a progressive transition from smooth, planktonic bacilli to complex three‐dimensional architectures with interwoven extracellular material and the appearance of pore‐like channels. This layered morphology suggests early spatial differentiation and matrix‐guided organization, consistent with biofilms formed directly on host tissues.

**FIGURE 2:**
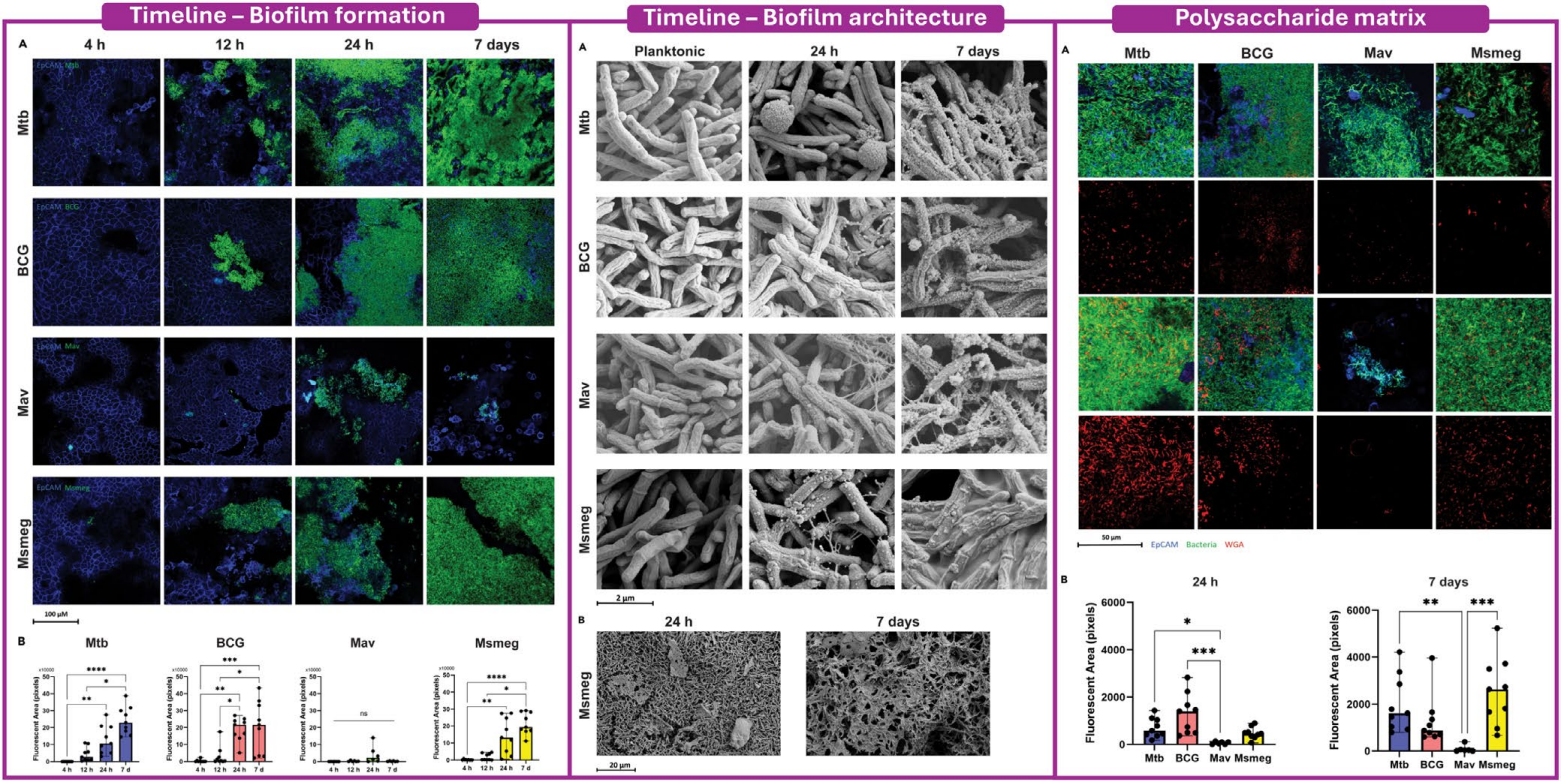
Early biofilm formation, ultrastructural maturation, and polysaccharide matrix development in pathogenic and non‐pathogenic mycobacteria. Left: Time‐resolved confocal microscopy showing the development of surface‐associated bacterial clusters on differentiated primary bronchial epithelial cells (PBEC) at 4 h, 12 h, 24 h, and 7 days for *Mycobacterium tuberculosis* (Mtb), *M. bovis* BCG, *M. avium* (Mav), and *M. smegmatis* (Msmeg). Bacteria are shown in green and epithelial cells (EpCAM) in blue. Quantification of bacterial fluorescent area over time is shown below. Middle: Scanning electron microscopy images illustrating structural differences between planktonic bacteria, early biofilm microcolonies (24 h), and mature 7‐day biofilms, revealing extracellular matrix accumulation and structural organization of the biofilm architecture. Right: Confocal microscopy analysis of extracellular polysaccharide matrix using wheat germ agglutinin (WGA) staining (red) in mycobacterial biofilms on PBEC at 24 h and 7 days, with corresponding quantification of WGA‐positive fluorescent area shown below. Figure adapted from Barclay et al. [128], iScience, published under the Creative Commons CC‐BY license, which permits reuse, distribution, and adaptation provided the original work is properly cited.

Matrix profiling using wheat germ agglutinin (WGA) indicates robust, species‐dependent accumulation of N‐acetylglucosamine–rich polysaccharides, particularly in Mtb, BCG, and *M smegmatis*, whereas *M. avium* generates more compact and heterogeneous structures. The matrix composition appears distinct from the cellulose‐rich TRS‐induced biofilms and from the lipid‐dense extracellular matrix described in pellicles, highlighting the existence of a third, epithelial‐specific biofilm phenotype.

A key advantage of the ALI–PBEC model is that biofilms emerge without chemical induction, immune lysates, or long‐term culture, enabling high‐resolution analysis of early adherence, spatial patterning, and matrix deposition in a host‐relevant microenvironment. This rapid formation also facilitates short‐timescale screening of antibiofilm compounds and the study of epithelial responses to structured bacterial communities. As such, epithelial‐associated biofilms complement the classical pellicle model by capturing the earliest extracellular events of Mtb–epithelium interaction and by providing a platform to interrogate biofilm initiation, matrix composition, and drug tolerance in a physiologically contextualized setting.

### 3.2 In Vivo Models

In vivo models are indispensable for tuberculosis research because they allow the investigation of Mtb infection within a fully integrated physiological and immunological context. These systems enable the study of host–pathogen interactions, immune responses, disease progression, and therapeutic efficacy under conditions that cannot be reproduced in vitro [12]. Murine models are the most widely used in TB research due to their genetic tractability, relatively low cost, and the availability of well‐established experimental protocols. Mice have been extensively employed to study innate and adaptive immune responses to Mtb, to dissect host genetic factors influencing susceptibility, and to evaluate vaccine candidates and antimicrobial regimens [12]. However, important differences exist between murine and human TB, particularly with respect to granuloma architecture, disease pathology, and metabolic responses, which limits the extent to which murine models fully reproduce the spectrum of human disease.

Non‐human primate models, especially macaques, are used to overcome some of these limitations. Due to their close phylogenetic relationship to humans, macaques develop pulmonary lesions, granuloma heterogeneity, latency, and reactivation patterns that more closely resemble human tuberculosis. As a result, these models are particularly valuable for studying TB pathogenesis, immune correlates of protection, latency, and the efficacy of vaccines and therapeutic interventions under clinically relevant conditions [129]. Guinea pigs represent another important in vivo model, characterized by their high susceptibility to Mtb infection. They develop progressive pulmonary disease with extensive tissue pathology, making them suitable for studying primary infection, disease progression, and the evaluation of antimicrobial efficacy, particularly in the context of bacterial burden reduction and lesion pathology [130].

More recently, zebrafish models have gained relevance as an in vivo system for TB research, primarily through the use of *Mycobacterium marinum as* a surrogate pathogen. Zebrafish offer unique advantages for real‐time visualization of granuloma formation, host–pathogen interactions, and immune cell dynamics in vivo. In addition, their compatibility with high‐throughput screening approaches makes them attractive for early‐stage drug discovery and mechanistic studies of granuloma biology [131]. Beyond their utility for studying granuloma dynamics, zebrafish infection models have recently been exploited to interrogate biofilm‐associated phenotypes and therapeutic responses in vivo. Using *M. marinum* as a surrogate, several studies have demonstrated that microbacterial aggregates exhibiting biofilm‐like properties persist within host tissues and display enhanced tolerance to antimicrobial treatment.

In a recent study [132], engineered mesoporous silica nanoparticles loaded with doxycycline showed potent activity against biofilm‐embedded *M. marinum* in zebrafish embryos, significantly reducing bacterial burden and improving host survival. Importantly, therapeutic efficacy correlated with disruption of structured bacterial communities rather than with effects on planktonic bacteria alone, providing in vivo evidence that biofilm‐associated tolerance constitutes a distinct and targetable phenotype.

Complementary in vivo and ex vivo analyses reported by Agu et al. [133] further support the relevance of mycobacterial aggregation and matrix‐associated persistence during host infection, revealing that mycolic acid–rich extracellular structures modulate host–pathogen interactions and antimicrobial accessibility. Together with recent work showing that biofilm‐like growth states of non‐tuberculous mycobacteria confer increased drug tolerance in animal models, these studies reinforce the translational importance of targeting biofilm‐associated phenotypes in tuberculosis research [107]. Although most contemporary in vivo biofilm studies rely on non‐tuberculous mycobacteria, the conserved nature of aggregation, matrix composition, and tolerance mechanisms supports their relevance for *M tuberculosis*, particularly in the context of granulomatous disease and persistent infection.

### 3.3 The Emergency of Computational Models and Artificial Intelligence

Computational models are increasingly incorporated into tuberculosis research as complementary tools to integrate experimental, clinical, and epidemiological data and to explore complex host–pathogen–drug interactions that are difficult to dissect experimentally. These approaches enable the simulation of infection dynamics, pharmacokinetics and pharmacodynamics, immune responses, and treatment outcomes, providing a systems‐level perspective that extends beyond isolated in vitro or in vivo models [134].

In the context of TB therapy, mathematical and computational frameworks have been used to model drug–drug interactions, optimize dosing regimens, and predict treatment endpoints, particularly for pulmonary disease. Comparative analyses of existing predictive models have highlighted both their potential to inform therapeutic strategies and their limitations, including variability in assumptions, data sources, and clinical applicability [135]. Importantly, these models increasingly aim to incorporate host‐specific parameters, such as immune status and metabolic environment, which are known to influence drug efficacy. For example, host‐driven modulation of pyrazinamide activity underscores the necessity of integrated models that explicitly account for host–pathogen interactions rather than bacterial factors alone [136].

Clinical and population‐level datasets further contribute to computational modeling efforts by providing real‐world evidence on treatment outcomes and disease modifiers. Systematic reviews and meta‐analyses examining vulnerable populations, including individuals experiencing homelessness, as well as comorbid conditions such as diabetes mellitus, have generated structured datasets that inform risk stratification and disease association models [137, 138, 139]. While these studies are not experimental models per se, they provide essential inputs for computational frameworks that seek to capture the heterogeneity of TB disease and treatment response.

Artificial intelligence–based approaches are beginning to complement traditional computational modeling by enabling the analysis of large, multidimensional datasets derived from imaging, transcriptomics, clinical records, and experimental platforms. In TB research, AI has been primarily explored for diagnostic support, outcome prediction, and data integration, rather than as a standalone modeling paradigm. At present, its principal value lies in enhancing pattern recognition and hypothesis generation from data produced by established in vitro and in vivo models, rather than replacing mechanistic experimentation [135, 140].

With respect to mycobacterial biofilms, computational and AI‐based approaches remain underdeveloped but conceptually important. A major limitation in advancing biofilm‐focused modeling in TB is the lack of validated, biofilm‐specific biomarkers that can be reliably detected in animal models or human samples. As highlighted previously, definitive demonstration of biofilm‐associated *M tuberculosis* phenotypes in vivo will require the identification of such markers and their integration into experimental and computational pipelines. As these data become available, computational models may play a critical role in linking biofilm‐associated physiology to drug tolerance, treatment failure, and disease persistence, thereby informing both therapeutic and diagnostic innovation [54, 112].

### 3.4 Mycobacteria Biofilms Triggers

Biofilm formation in mycobacteria, including Mtb, constitutes a regulated adaptive strategy that promotes long‐term survival, persistence, and phenotypic drug tolerance rather than a passive aggregation state. Accumulating evidence indicates that biofilm development is driven by defined environmental cues, genetic determinants, and regulatory networks that collectively shape biofilm architecture, metabolic organization, and antibiotic responsiveness over time [11].

Environmental and physicochemical signals play a central role in initiating and modulating mycobacterial biofilm formation. Nutrient limitation, oxygen availability—particularly hypoxia—redox imbalance, acidic pH, temperature shifts, and fluctuations in ion concentrations are among the most relevant triggers, many of which are encountered during infection and within host tissues [141]. These conditions not only promote biofilm initiation but also influence matrix composition, spatial organization, and the emergence of drug‐tolerant subpopulations.

Adaptation to these environmental stresses requires coordinated regulatory responses. In Mtb, the sensor kinase MtrB functions as a key environmental sensor that enables bacterial adaptation to hypoxia, acidic pH, and nutrient deprivation. MtrB interacts with the DosR dormancy regulon, enhancing DosR binding to promoters of stress‐responsive genes such as hspX, thereby supporting survival of aggregated bacteria under low‐oxygen conditions and promoting biofilm‐associated persistence within host environments [142]. These regulatory circuits illustrate how classical dormancy pathways intersect with biofilm development rather than represent independent survival strategies.

Reductive stress represents another potent trigger of biofilm formation. Under thiol reductive stress conditions, Mtb displays a coordinated transcriptional response characterized by induction of stress‐responsive sigma factors such as sigE and sigB, along with redox‐associated regulators including whiB3; although their precise contributions to biofilm maturation remain incompletely defined. Concurrent upregulation of the ESX‐3 type VII secretion system and iron‐responsive regulators such as furA highlights a critical requirement for iron acquisition during biofilm growth. Additional transcriptional changes affecting cysteine and arginine metabolism, together with overexpression of the SenX3/RegX3 two‐component system and the serine/threonine kinase pknA, further support the existence of oxygen and nutrient gradients within the biofilm and underscore the regulatory complexity of this lifestyle [112].

Metabolic reprogramming is a defining feature of mycobacterial biofilms and directly contributes to phenotypic drug tolerance. Patil and Jain demonstrated that Mtb forms persister‐like biofilms composed of non‐replicating yet metabolically active cells that undergo a pronounced trehalose metabolic shift. In this state, trehalose utilization is redirected away from the synthesis of surface glycolipids such as trehalose monomycolate and trehalose dimycolate toward central carbon metabolism, including glycolysis and the pentose phosphate pathway. This reprogramming enhances ATP production, NADPH generation, and antioxidant capacity, thereby enabling survival under antibiotic stress. The trehalose synthase TreS plays a critical role in this process by modulating maltose production and downstream metabolism, directly influencing persister formation, biofilm stability, and drug tolerance [143].

Genetic analyses have further reinforced that biofilm formation in Mtb is a genetically programmed and metabolically distinct state. Genome‐wide screens identified approximately 53 genes as essential for biofilm growth, including phoT, pstC2A1, and dgt, which are involved in phosphate transport and nucleotide metabolism, as well as Rv0097, Rv0188, Rv0502, Rv1692, Rv3150, Rv3484, and Rv3514, which contribute to cell envelope maintenance, redox balance, and stress adaptation. Mutants lacking phoT or pstC2A1 exhibit defective biofilm formation and increased antibiotic susceptibility, establishing a direct genetic link between biofilm fitness and phenotypic drug tolerance [115]. Transcriptomic and lipidomic profiling further revealed selective induction of the isonitrile lipopeptide (INLP) operon (Rv0096–Rv0101) in biofilm cultures, defining a biofilm‐specific metabolic program distinct from planktonic growth.

Computational and network‐based analyses have expanded this framework by predicting additional regulators involved in biofilm formation, including Rv0081, DevR, RegX3, Rv0097, and Rv1996. Many of these factors are conserved across biofilm‐forming mycobacterial species, supporting the concept that biofilm formation represents an evolutionarily conserved lifestyle rather than an incidental response to stress [144].

Structural components of the mycobacterial cell envelope also act as critical triggers of biofilm development. Keto‐mycolic acids have been identified as essential molecular determinants of pellicle biofilm formation in Mtb. Sambandan et al. demonstrated that mutants lacking mmaA4 and therefore deficient in keto‐mycolic acid synthesis are unable to form pellicle biofilms under air–liquid interface conditions in Sauton's medium lacking Tween 80. Notably, co‐culture of ΔmmaA4 mutants with wild‐type bacteria restored protection against rifampicin killing, indicating that pellicle architecture itself confers drug tolerance independently of intrinsic resistance mechanisms. The absence of surfactants and the presence of an air–liquid interface thus function as permissive triggers that enable lipid‐mediated aggregation and matrix stabilization [113].

Redox signaling and metal availability further modulate biofilm formation across mycobacterial species. In *M. avium* , exposure to the quorum‐sensing molecule AI‐2 or to hydrogen peroxide enhances biofilm formation, suggesting that oxidative stress acts as a conserved biofilm‐inducing signal [145]. Iron availability is another key determinant: transcriptomic analyses in *M smegmatis* revealed strong induction of iron‐responsive genes, including those involved in siderophore synthesis and iron uptake, during biofilm development, even under moderate iron supplementation. In Mtb, reduction of iron concentrations or omission of supplemental zinc severely impairs biofilm maturation while leaving planktonic growth largely unaffected, highlighting a specific requirement for metal homeostasis during biofilm development and late‐stage matrix maturation [146].

### 3.5 Biofilm Morphology and Composition in Mycobacteria

#### 3.5.1 Architecture and Extracellular Matrix Composition of Mtb Biofilms

Biofilms formed by Mtb are not amorphous extracellular layers but highly organized, multicellular structures in which bacterial cells are embedded within a self‐produced extracellular matrix. This structured organization confers a substantial protective advantage against antimycobacterial drugs and host immune defenses and is increasingly recognized as a defining feature of persistent infection [104]. Biofilm‐associated growth in Mtb is closely linked to characteristic morphological phenotypes, most notably cording, which has long been associated with virulence and pathogenicity and is now understood to be reinforced within structured multicellular aggregates [147, 148]. Within host tissues, particularly in necrotic lung lesions, hypoxic and nutrient‐limited conditions further stabilize this slow‐metabolizing, persistent state that is characteristic of biofilm physiology [149].

Morphologically, mycobacterial biofilms are typically described as thick, rugose, and densely packed three‐dimensional structures. Across different experimental models, these biofilms display pronounced surface wrinkling and internal heterogeneity, features that are thought to enhance resilience by promoting spatial segregation and metabolic differentiation within the community [150]. Various in vitro and ex vivo biofilm models generate complex architectures that can be readily visualized using confocal laser scanning microscopy (CLSM) and scanning electron microscopy (SEM), revealing tightly packed bacterial aggregates interspersed with channels and voids that facilitate nutrient diffusion and waste removal [151]. These architectural features are consistent with observations made in host‐associated settings, where clustered bacilli organized within extracellular material have been detected inside necrotic lesions and granulomatous structures in infected lungs [152, 153].

In both in vitro pellicle models and in vivo infection contexts, Mtb biofilms manifest as structured multicellular assemblies rather than dispersed bacterial populations. Pellicles formed at the air–liquid interface recapitulate several features observed in necrotic lung tissue, including dense aggregation, lipid‐rich extracellular material, and marked drug tolerance [152, 153, 154, 155]. Within host lungs, these biofilm‐like clusters create protected microenvironments that promote immune evasion and persistence, contributing to treatment failure and relapse [152, 153].

The identification and characterization of Mtb biofilms rely on a combination of structural, chemical, and spectroscopic approaches. Among the most informative biomarkers are cellulose and free mycolic acids, which serve as hallmarks of the biofilm extracellular matrix. Cellulose has been detected using Calcofluor White staining, fluorescent cellulose‐binding probes such as CBD–mCherry, DNS‐based biochemical assays, and Raman microscopy, providing both qualitative and quantitative evidence of polysaccharide‐rich matrices in Mtb biofilms [156]. Importantly, cellulose‐rich material has been visualized not only in vitro but also in lung tissues from experimentally infected mice, non‐human primates, and human tuberculosis patients, establishing its relevance in vivo [122, 157].

Free mycolic acids represent a second major biomarker of biofilm‐associated growth. These lipids are readily distinguished from cell‐wall‐bound mycolic acids using two‐dimensional thin‐layer chromatography (TLC), nuclear magnetic resonance (NMR) spectroscopy, and MALDI‐TOF mass spectrometry. Quantitative analyses have shown that free mycolic acids can be up to fivefold more abundant in biofilm cultures than in planktonic Mtb populations and are associated with a substantial subpopulation of drug‐tolerant persister cells, estimated to represent approximately 8%–10% of the biofilm community [158]. The enrichment of these lipid species correlates with mature biofilm architecture and provides a robust biochemical signature distinguishing biofilm‐grown Mtb from free‐living cells.

Together, these morphological, architectural, and analytical observations establish Mtb biofilms as structured, matrix‐embedded communities that can be reliably detected and characterized using complementary imaging and biochemical techniques. The convergence of rugose morphology, cording, polysaccharide‐ and lipid‐rich matrices, and spatially organized aggregates within host tissues underscores the biological relevance of biofilm‐associated growth and provides a framework for linking structure to persistence and drug tolerance.

Beyond their characteristic architecture, mycobacterial biofilms are defined by a chemically complex EPS matrix that encases bacterial cells and determines both mechanical stability and functional behavior. In Mtb, this matrix is composed of polysaccharides, lipids, proteins, and extracellular DNA (eDNA), whose relative contributions differ from those observed in classical Gram‐negative biofilms and confer unique physicochemical properties to the community [105, 159, 160].

Among these components, cellulose has emerged as a central structural scaffold of the Mtb biofilm matrix. Multiple studies have demonstrated that cellulose‐rich material surrounds biofilm‐grown bacilli, providing rigidity and cohesion to multicellular aggregates [161]. Functional relevance of cellulose was established by genetic and enzymatic perturbation experiments showing that disruption of cellulose synthesis or enhanced cellulase activity leads to defective biofilm formation and reduced persistence. Notably, Mtb strains engineered to overexpress cellulase enzymes exhibited impaired biofilm development and attenuated survival in murine infection models, directly linking cellulose‐containing matrices to virulence and in vivo fitness [156]. The presence of cellulose has been conclusively confirmed not only in in vitro biofilms but also in lung tissues from infected mice, non‐human primates, and human tuberculosis patients, where bacilli appear embedded within polysaccharide‐rich extracellular material [122, 157]. Comparable polysaccharide matrices have been described in non‐tuberculous mycobacteria, where α‐D‐glucopyranosyl and mannopyranosyl residues predominate, underscoring a conserved role for carbohydrate scaffolds across mycobacterial biofilms [162].

Lipids constitute a second major component of the mycobacterial biofilm matrix and are particularly enriched in biofilm‐grown Mtb. Free mycolic acids represent the dominant lipid species and are markedly more abundant in biofilms than in planktonic cultures [136, 158, 163, 164]. Unlike cell wall–anchored mycolic acids, these free lipids are secreted into the extracellular space, where they contribute to hydrophobic interactions, matrix cohesion, and pellicle formation at the air–liquid interface. Lipid biosynthesis underlying biofilm formation is tightly linked to the FAS‐II pathway, and mutations affecting this system result in defective pellicle development. Chromatographic analyses identified a lipid species originally designated “Spot 1” as a mixture of free mycolic acids, including methoxy mycolates and α‐mycolates, with minor contributions from keto mycolates, a profile characteristic of mature Mtb biofilms [165, 166]. The selective accumulation of free mycolic acids distinguishes biofilm‐grown Mtb from planktonic populations and correlates with the emergence of drug‐tolerant persister subpopulations [167, 168].

Additional lipid species further contribute to matrix organization in mycobacteria. Meromycolyl diacylglycerols and glycopeptidolipids have been implicated in biofilm formation and surface interactions across several mycobacterial species, reinforcing the concept that lipid diversity plays a central role in shaping biofilm architecture and stability [148, 169, 170].

Proteins within the biofilm matrix fulfill both structural and regulatory functions. Protease sensitivity assays demonstrate that protein components are required to maintain matrix integrity, as treatment with proteinase K leads to partial or complete biofilm disassembly in both Mtb and non‐tuberculous mycobacteria [171, 172, 173]. Comparative proteomic analyses have revealed selective enrichment of stress‐responsive proteins in biofilm‐grown Mtb, with HspX emerging as one of the most consistently upregulated factors. Elevated HspX expression reflects adaptation to hypoxia and nutrient limitation and is thought to support biofilm‐associated persistence and drug tolerance [153, 174, 175]. In addition to stress proteins, enzymatic components contribute to matrix remodeling. Mtb‐encoded cellulases, including Rv0062 and Rv1090, can degrade cellulose within the matrix, suggesting that biofilm architecture is dynamically regulated rather than static [176].

Perturbation of protein‐mediated metabolic pathways further highlights their importance for biofilm integrity. Deletion of ppk1 in *Mycobacterium smegmatis* results in severe polyphosphate depletion, leading to sparse, poorly cohesive biofilms accompanied by altered lipid composition and increased sensitivity to antibiotics such as vancomycin, linking energy storage and envelope homeostasis to biofilm maturation and persistence [150].

Overexpression of protein *O*‐mannosyltransferase (PMT; Rv1002c) in both Mtb and *M smegmatis* enhances biofilm formation and suppresses pro‐inflammatory cytokine release, indicating that protein glycosylation modulates not only matrix assembly but also host immune responses in favor of bacterial survival [177]. Similarly, the cyclophilin peptidyl‐prolyl isomerase PpiB promotes pellicle and biofilm formation in *M smegmatis* and Mtb H37Rv, further underscoring the contribution of protein folding and stress adaptation pathways to biofilm development [178].

The enzyme GlmU provides an additional link between stress sensing, cell wall metabolism, and biofilm formation. In *M smegmatis*, GlmU is induced under DNA alkylation stress and enhances tolerance to environmental insults and antibiotics by promoting biofilm formation. Given its role in the synthesis of UDP‐*N*‐acetylglucosamine, a key precursor for peptidoglycan and related structures, GlmU represents a mechanistic bridge between cell envelope biosynthesis and biofilm‐associated persistence [179].

Extracellular DNA constitutes a further component of the mycobacterial biofilm matrix, although its relative importance varies among species. In non‐tuberculous mycobacteria, eDNA has been visualized within the matrix and contributes to aggregation, but DNase I treatment often produces only modest disruption compared with enzymatic degradation of cellulose or proteins [180, 181]. Within host lesions, both bacterial and host‐derived DNA may integrate into the extracellular matrix, potentially impairing phagocytosis and shielding embedded bacilli from extracellular immune killing mechanisms [182].

Recent studies extended these observations to slow‐growing mycobacteria, including Mtb, *M. intracellulare* , and *M. avium* , demonstrating that eDNA is present during exponential growth and contributes to pellicle and floating biofilm formation. Removal of eDNA significantly reduces biofilm biomass and increases susceptibility to antibiotics such as isoniazid and amikacin, highlighting eDNA as a modifiable matrix component with therapeutic relevance [180, 183]. Taken together, the composition of the mycobacterial biofilm matrix reflects a highly coordinated assembly of polysaccharides, lipids, proteins, and nucleic acids that collectively define a distinct physiological state. The enrichment of cellulose and free mycolic acids provides mechanical stability and hydrophobic shielding, while protein‐ and DNA‐mediated interactions enable dynamic remodeling and stress adaptation. This composite matrix not only underpins biofilm architecture but also facilitates the emergence of drug‐tolerant persister populations and long‐term survival within host tissues, establishing biofilm composition as a central determinant of mycobacterial persistence and a rational target for therapeutic intervention.

#### 3.5.2 Biofilm Formation in Non‐Tuberculous Mycobacteria

**FIGURE 3:**
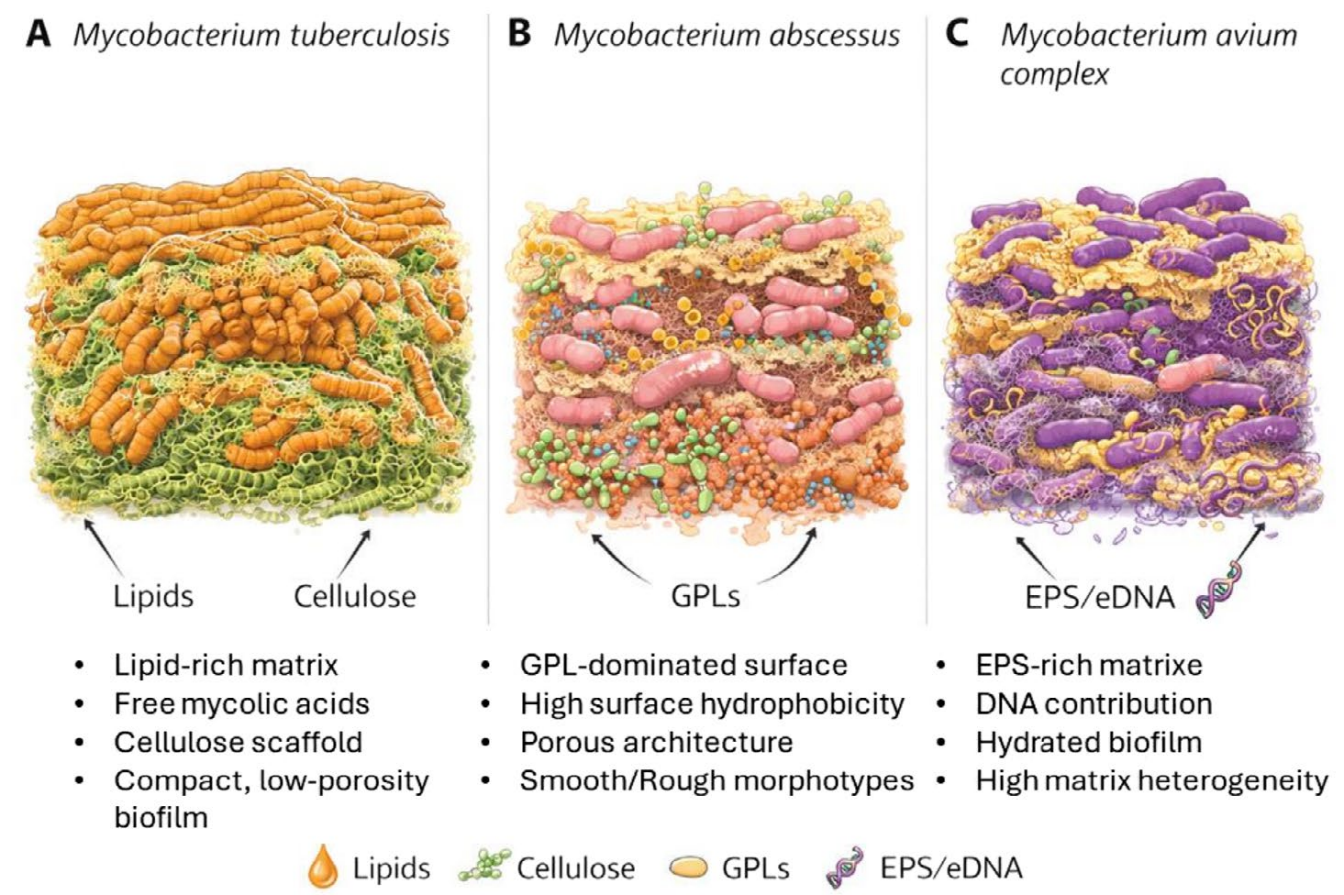
Conceptual representation of species‐specific biofilm architectures in pathogenic mycobacteria. Schematic illustration summarizing experimentally reported differences in extracellular matrix composition and structural organization of biofilms formed by *Mycobacterium tuberculosis* , rapidly growing non‐tuberculous mycobacteria ( *M. abscessus* ), and members of the *Mycobacterium avium* complex. The figure highlights lipid‐ and cellulose‐enriched matrices in *M. tuberculosis* , GPL‐dominated surface architectures in rapidly growing mycobacteria, and polysaccharide‐ and eDNA‐rich matrices in MAC species. This illustration is a conceptual synthesis based on published experimental studies and does not represent direct microscopy data.

Biofilm formation in NTM is governed by molecular determinants that differ substantially from those characterized in Mtb, resulting in species‐specific ECM architectures and tolerance phenotypes [184]. A conceptual summary of the major differences in biofilm architecture and dominant extracellular matrix components across pathogenic mycobacterial species is provided in Figure 3. In rapidly growing NT, including *M. abscessus, M. fortuitum* and *M. chelonae*, biofilm initiation and surface attachment are primarily dictated by the abundance and chemical composition of glycopeptidolipids (GPLs) in the outer cell envelope [185]. GPLs directly control surface hydrophobicity, sliding motility and cell–cell cohesion, thereby determining early biofilm morphology and stability. In *M. abscessus* , loss or modification of GPLs drives the transition from smooth to rough morphotypes, a switch associated with reduced surface spreading, increased aggregation, and enhanced invasiveness [186]. Rough variants form dense, compact aggregates with limited surface organization, whereas smooth variants generate flatter, surface‐attached biofilms with defined boundaries. This GPL‐dependent biofilm program is absent in Mtb, which lacks GPLs and relies on lipid remodeling rather than surface glycolipids to sustain extracellular growth [86].

In Mtb, biofilm maturation is linked to selective enrichment of free mycolic acids, particularly keto‐mycolates, within the extracellular matrix, producing hydrophobic pellicle structures at the air–liquid interface. By contrast, lipidomic analyses in *M. abscessus* biofilms reveal remodeling of α‐mycolic acid subclasses and accumulation of free mycolic acids that differ in chain length distribution and relative abundance from those observed in Mtb [117]. These differences translate into distinct ECM packing densities and permeability properties, as reflected by the more porous and filamentous pellicle surfaces observed in *M. abscessus* compared with the compact, lipid‐dense pellicles typical of Mtb. Thus, while lipid enrichment is a conserved feature of mycobacterial biofilms, the specific lipid species and their spatial organization are species‐dependent [117, 185].

Biofilms formed by members of the *Mycobacterium avium* complex (MAC) exhibit a third organizational pattern. In *M. avium* and *M. intracellulare* , the extracellular matrix contains a higher proportion of extracellular polymeric substances, including polysaccharides and eDNA [120, 180]. DNase‐sensitive matrix components have been repeatedly detected in MAC biofilms, indicating a structural role for eDNA in cohesion and biofilm stability. Although eDNA is also detectable in biofilms of Mtb and rapidly growing NTM, its relative contribution differs: in MAC it functions as a major structural element, whereas in Mtb it complements a matrix dominated by lipids and cellulose, and in rapidly growing NTM it integrates into GPL‐centered assemblies [180]. These compositional differences directly affect biofilm hydration, mechanical resilience, and antibiotic penetration.

At the regulatory level, partial conservation is observed alongside functional divergence. The chaperone GroEL1, essential for biofilm maturation in Mtb through its role in mycolic acid biosynthesis and redox balance, is conserved across mycobacteria [187]. However, in NTM, GroEL1 activity is embedded within regulatory contexts shaped by GPL metabolism and polysaccharide‐rich matrices rather than by lipid‐driven pellicle formation. Consequently, disruption of GroEL1‐dependent pathways produces distinct phenotypic outcomes in NTM compared with Mtb, underscoring that shared regulators do not imply conserved biofilm architectures [120, 180].

These molecular differences translate into distinct tolerance phenotypes, with NTM biofilms formed in aquatic environments and on biomaterials exhibiting high tolerance to macrolides, aminoglycosides, and β‐lactams as a consequence of matrix composition, metabolic heterogeneity, and envelope remodeling rather than classical resistance mutations [184, 188]. In contrast, biofilm‐associated tolerance in Mtb is closely linked to metabolic downshifts, redox adaptation and lipid‐mediated diffusion barriers under host‐mimicking conditions. As a result, antibiofilm strategies validated in NTM—such as targeting GPL‐mediated adhesion or eDNA‐rich matrices—cannot be directly extrapolated to Mtb without mechanistic validation [185]. Biofilm formation in NTM is governed by molecular determinants that differ substantially from those characterized in Mtb, resulting in species‐specific ECM architectures and tolerance phenotypes (Table 3).

**TABLE 3:** Comparative features of biofilm composition and architecture across mycobacterial species [120, 189, 190, 191, 192, 193, 194, 195].

Feature	*Mycobacterium tuberculosis*	*Mycobacterium abscessus*	*Mycobacterium avium* complex (MAC)	Other rapidly growing NTM ( *M. fortuitum* , *M. chelonae* )
Primary biofilm context	Host‐associated, hypoxic, nutrient‐limited microenvironments	Abiotic surfaces, airways, biomaterials	Environmental reservoirs, water systems, and host tissues	Environmental and device‐associated surfaces
Dominant envelope determinants	Free mycolic acids, mycolic acid remodeling	Glycopeptidolipids (GPLs)	Mixed lipid–polysaccharide envelope	GPL‐rich outer envelope
Role of GPLs	Absent or negligible	Central regulator of adhesion, aggregation, and morphotype switching	Present but less dominant than in *M. abscessus*	Major contributor to surface hydrophobicity
Extracellular DNA (eDNA)	Detected; contributes to matrix cohesion	Detected; contributes to biofilm stability	Detected; structural role in matrix	Likely present, less well characterized
Polysaccharide‐rich matrix	Limited, lipid‐dominated matrix	Variable; secondary to lipid components	Prominent component of the extracellular matrix	Variable, species‐dependent
Key protein regulators	GroEL1, redox‐associated proteins	GroEL1 and envelope‐associated factors	Stress‐response and envelope proteins	Poorly defined
Biofilm architecture	Pellicle‐like, lipid‐rich aggregates	Surface‐attached, morphotype‐dependent (smooth vs. rough)	Structured, hydrated biofilms	Surface‐associated aggregates
Metabolic state within biofilm	Strongly reduced, non‐replicating persistence	Heterogeneous; active peripheral cells	Reduced metabolic activity	Heterogeneous
Primary tolerance mechanisms	Metabolic dormancy, envelope remodeling	Matrix protection, envelope adaptation	Matrix‐mediated protection, slow growth	Envelope‐mediated tolerance
Relevance to antimicrobial tolerance	High tolerance under host‐like conditions	Marked tolerance to multiple antibiotics	High tolerance in chronic and device‐related infections	Clinically relevant tolerance
Clinical implication	Persistence during long‐term TB therapy	Recalcitrant pulmonary and device infections	Chronic infection requiring prolonged therapy	Difficult‐to‐eradicate environmental infections

### 3.6 Drug Resistance in Mycobacteria in Biofilms

Biofilm‐mediated tolerance in mycobacteria arises from the convergence of multiple, partially overlapping [115, 196, 197]. A primary contributor is the extracellular matrix, enriched in structural polysaccharides such as cellulose and lipid components, including free mycolic acids, which collectively restrict the diffusion and penetration of antimicrobial agents into the biofilm interior [142, 198]. This physical barrier reduces local drug concentrations and limits bactericidal activity, particularly against cells embedded in deeper biofilm layers. In parallel, biofilm‐associated mycobacteria undergo profound metabolic reprogramming. Cells within biofilms shift central metabolic pathways, notably trehalose metabolism, to sustain intracellular ATP levels and redox balance through the maintenance of antioxidant capacity. These adaptations support slow growth or dormancy and contribute to phenotypic drug tolerance rather than heritable resistance [148, 199, 200].

Stress response pathways further reinforce biofilm‐associated tolerance. Induction of stress‐responsive regulons enhances survival under antibiotic pressure by promoting damage repair, redox homeostasis, and adaptation to nutrient limitation, hypoxia, and oxidative stress [201, 202]. In addition, the upregulation of efflux pumps and other stress‐related genes reduces intracellular drug accumulation and contributes to a multidimensional tolerance phenotype that is difficult to eradicate with conventional therapies [203, 204]. Experimental studies consistently demonstrate that bacilli residing within mycobacterial biofilms survive concentrations of isoniazid and rifampicin that are lethal to planktonic populations. These findings support the conclusion that a substantial fraction of drug recalcitrance in Mtb arises from biofilm‐associated phenotypic tolerance rather than classical genetic resistance [197]. Consequently, standard planktonic susceptibility assays are likely to underestimate clinically relevant tolerance mechanisms, contributing to prolonged treatment durations, increased relapse risk, and the persistent discrepancy between in vitro drug potency and sterilizing activity observed in patients.

The clinical relevance of mycobacterial biofilms is further strengthened by evidence demonstrating that Mtb and related species form structured biofilms directly on host epithelial surfaces. Using primary human bronchial epithelial cells, Barclay et al. showed that *M tuberculosis* rapidly establishes polysaccharide‐rich biofilms within 24 h, with progressive matrix accumulation and architectural maturation over time. These epithelial‐associated biofilms exhibit dense extracellular matrices containing polysaccharides and lipid components, including free mycolic acids, which spatially encapsulate bacilli and generate heterogeneous microenvironments that limit antibiotic penetration. Notably, bacilli embedded within these biofilms survive concentrations of isoniazid and rifampicin that are lethal to planktonic populations, providing direct experimental evidence that biofilm growth on host tissues promotes phenotypic drug tolerance rather than classical genetic resistance [128].

Mechanistic insight into the matrix‐dependent nature of this tolerance is provided by genetic dissection of biofilm architecture in *M. marinum* , a close relative of *M tuberculosis*. Deletion of the putative glycosyltransferase epsH resulted in excessive accumulation of extracellular matrix components, particularly extracellular DNA and proteins, leading to increased antibiotic tolerance in vivo despite variable effects under in vitro conditions. Strikingly, in an adult zebrafish infection model, ΔepsH biofilms displayed markedly reduced responsiveness to rifampicin–moxifloxacin treatment compared with wild‐type infections, underscoring the dominant protective role of the biofilm matrix in vivo. These findings highlight that biofilm‐mediated tolerance emerges from the integrated effects of matrix composition, growth dynamics, and host‐associated conditions, and emphasize that targeting extracellular matrix integrity represents a promising strategy to overcome drug recalcitrance in mycobacterial infections [205].

### 3.7 Immunological Landscape

Pulmonary infection by Mtb induces the formation of granulomatous lesions within lung tissue, which represent the hallmark immunopathological feature of tuberculosis. Following inhalation of Mtb‐containing aerosols, granulomas begin to develop within alveolar macrophages approximately four weeks post‐infection. These early or primary granulomas progressively mature into more complex immune structures over time [86]. Structurally, granulomas are compact immune aggregates composed predominantly of macrophages at the core, many of which differentiate into foamy macrophages and multinucleated giant cells enriched in lipid droplets. This central macrophage‐rich region is surrounded by concentric layers of T and B lymphocytes, forming an organized immune microenvironment [152].

Within tuberculosis granulomas, particularly at peripheral regions adjacent to lymphocyte‐rich zones, Mtb bacilli can organize into biofilm‐like aggregates embedded within an extracellular matrix. These structured bacterial assemblies interfere with the activity of first‐line anti‐tuberculosis drugs such as isoniazid and rifampicin, limit immune cell access, and serve as reservoirs for drug‐tolerant persister populations that sustain chronic infection [206]. The spatial localization of these biofilm‐like aggregates within immune niches suggests a close interplay between bacterial organization and host immune pressure.

Biofilm and pellicle formation within pulmonary cavities further contribute to tuberculosis pathogenesis. These structures have been implicated in caseous necrosis, cavitation, and long‐term bacterial persistence, as well as in the development of phenotypic drug tolerance [116]. During transmission, aerosols expelled from infected individuals may contain bacilli released from pellicle‐like biofilms lining lung cavities. Such bacilli may be transiently protected within extracellular matrices, as proposed, enhancing environmental survival and shielding them from immediate immune recognition [207]. The capacity of clinical Mtb isolates to robustly form biofilms supports the notion that this phenotype plays a critical role in disease persistence and propagation, including during secondary tuberculosis [86].

The immunological milieu within granulomas strongly influences biofilm establishment and maintenance. Several cytokines associated with chronic inflammation and granuloma stability—including IL‐23, IL‐21, IL‐27, IL‐22, and IL‐18—have been shown to shape immune environments that are permissive to bacterial extracellular matrix production and long‐term persistence [208]. In latent tuberculosis, a balanced coexistence of pro‐inflammatory and regulatory cytokines may limit bacterial clearance while permitting survival within biofilm‐like communities [209]. This immunomodulatory equilibrium promotes metabolic heterogeneity among bacterial populations and enhances tolerance to both immune‐mediated killing and antimicrobial treatment.

Biofilm growth in Mtb is accompanied by specific modifications in cell wall carbohydrate composition, including altered proportions of glucose‐, mannose‐, and arabinose‐containing residues. These changes are associated with reduced activation of the classical and lectin complement pathways, as reflected by decreased C3b/iC3b deposition on biofilm‐derived carbohydrate extracts [104]. Such modifications contribute to immune evasion by diminishing opsonization and complement‐mediated clearance.

Several Mtb proteins expressed during biofilm growth directly modulate host immune signaling. The protein PPE10 enhances bacterial tolerance to oxidative and acidic stress and promotes intracellular persistence by targeting the host linear ubiquitin chain assembly complex (LUBAC), a central regulator of NF‐κB–mediated inflammatory signaling. By interfering with LUBAC activity, PPE10 suppresses NF‐κB activation, reduces production of pro‐inflammatory cytokines such as IL‐1β, IL‐6, and TNF‐α, and limits caspase activation, thereby prolonging host cell survival and stabilizing the intracellular niche supporting biofilm maintenance [210].

Alterations in mycolic acid biosynthesis further link biofilm structure to immune interactions. In *M. smegmatis* mutants lacking MSMEG4722, which encodes a reductase required for late‐stage mycolic acid synthesis, biofilms display reduced mass and impaired accumulation of mature free mycolic acids [163]. In these mutants, enhanced deposition of the complement opsonin C3b is observed on planktonic bacteria, whereas biofilm‐associated cells remain comparatively protected. These findings indicate that mycolic acid maturation is critical for proper biofilm development and modulates complement‐mediated immune recognition [148, 211].

Additional immunomodulatory mechanisms involve the biofilm‐associated expression of PE31 (Rv3477). Experimental studies using recombinant *M smegmatis* expressing PE31 demonstrated induction of guanylate‐binding protein 1 (GBP‐1) in macrophages, leading to reduced caspase‐3 activation and suppression of apoptosis. This prolongation of host cell viability creates a permissive intracellular environment that supports biofilm persistence [171]. PE31 expression is also associated with altered cytokine profiles, characterized by reduced levels of IL‐12p40 and IL‐6 and increased IL‐10 production, further dampening host immune responses and facilitating long‐term bacterial survival in biofilm‐associated states.

## 4 Strategies to Disrupt Biofilms in Mycobacteria

The persistent and highly drug‐tolerant nature of mycobacterial biofilms has intensified the need for innovative therapeutic routes that move beyond conventional antibiotic monotherapy [45, 212]. In response, recent research has advanced three complementary strategy domains: antibiotic–antibiotic synergy, peptide–antibiotic combinations and nanotechnology‐based drug delivery systems. Together, these approaches not only enhance bactericidal activity but also directly target the structural and functional properties that underlie biofilm‐associated tolerance in *Mycobacteria*.

### 4.1 Antibiotic–Antibiotic Combinations

A triple‐agent strategy incorporating amikacin, gatifloxacin, and deoxyribonuclease (DNase) achieved enhanced antimicrobial activity through a dual mechanism: direct bacterial killing by the antibiotic combination and disruption of the biofilm extracellular matrix by enzymatic degradation of eDNA [213]. This synergistic approach demonstrated significant efficacy both in vitro against mycobacterial biofilms and in vivo in animal models of mycobacterial keratitis, representing a paradigm shift by simultaneously targeting bacterial viability and biofilm structural integrity. The DNase component specifically addresses the protective barrier function of eDNA within the biofilm matrix, thereby enhancing antibiotic penetration and access to embedded bacteria that would otherwise remain protected from antimicrobial agents.

The combination of the antimycobacterial agents bedaquiline and clofazimine represents an option for difficult‐to‐treat NTM lung disease, including *M. abscessus* infections [214]. The efficacy stems from complementary mechanisms: bedaquiline inhibits mycobacterial ATP synthase while clofazimine disrupts membrane function and exhibits anti‐biofilm properties. Clinical and experimental reports have documented the activity of this combination against biofilm‐forming NTM isolates. In vitro studies demonstrate that synergy enhances bacterial killing within biofilm matrices, overcoming the tolerance typically observed with monotherapy. In refractory patients who failed conventional treatment regimens—often including macrolides (azithromycin or clarithromycin), aminoglycosides (amikacin), and β‐lactams (cefoxitin or imipenem)—this combination has demonstrated clinical benefit, offering a therapeutic option when traditional antimycobacterial agents prove ineffective.

A recent study evaluated the anti‐biofilm activities and antibiotic synergy of 12 naturally occurring compounds against drug‐resistant RGM, including *M. abscessus* , *M. fortuitum* , and *M. chelonae* [215]. Four compounds—trans‐cinnamaldehyde, carvacrol, gentisaldehyde, and phloroglucinaldehyde—showed strong growth inhibition and markedly reduced biofilm formation (to 2.9%–20.5% of controls). Checkerboard assays revealed significant synergy between these compounds and key antibiotics (amikacin, clarithromycin, linezolid), with combinations such as carvacrol–linezolid, carvacrol–amikacin, and gentisaldehyde–clarithromycin showing the strongest effects. The work highlights the promise of essential oils and phenolic aldehydes as standalone or combination therapies against resistant RGM and underscores their potential for environmental applications targeting microbial persistence.

### 4.2 Peptide–Antibiotic Synergy

Members of the LAK antimicrobial peptide family act by disrupting mycobacterial aggregation through detergent‐like effects, which prevent bacterial cell clumping in inhalable formulations [216]. These peptides were tested in vitro against MDR and XDR *M tuberculosis* strains, particularly in combination with isoniazid [217]. The combinations achieved strong synergy, with FICI values ranging from 0.25 to 0.38. Such findings provide compelling evidence that inhaled AMP–antibiotic regimens could serve as powerful adjunct therapies in tuberculosis or tuberculosis‐related diseases, especially in cases where resistance severely limits treatment options [218, 219].

Li et al. investigated the synthetic antimicrobial peptide RP557 and found that it was effective at weakening the structural integrity of *M. abscessus* biofilms [220]. Through SEM imaging and viability staining, the team showed how RP557 physically disrupted the dense biofilm matrix and suppressed genes linked to biofilm persistence and metabolic activity. What particularized this work was the demonstration that once the biofilm was destabilized, *M. abscessus* became far more vulnerable to conventional antibiotics—clarithromycin, amikacin, cefoxitin, and imipenem all exhibited greatly enhanced activity when paired with RP557. The study included direct Minimum Biofilm Eradication Concentration and viability data, highlighting the strong potential of peptide–antibiotic combinations for treating notoriously drug‐tolerant *M. abscessus* infections.

In another research, the ApoE‐mimetic peptide COG1410 was explored using *M smegmatis* as a model system [221]. Beyond simply killing planktonic cells, COG1410 was able to block biofilm formation altogether and, when combined with standard antibiotics (azithromycin, amikacin, linezolid, ciprofloxacin, and cefoxitin), it frequently produced additive or even synergistic effects. It was revealed that the peptide interacts with the bacterial protein ClpC, suggesting a non‐traditional mode of action that goes beyond the membrane disruption typically associated with antimicrobial peptides. This study underscores how mechanistically diverse peptides like COG1410 can be paired with antibiotics to expand our anti‐mycobacterial arsenal.

### 4.3 Drug Delivery Systems‐Based Nanotechnology Pathways

Pati et al. engineered transferrin‐conjugated silver quantum dots encapsulating a Zn–rifampicin complex to promote targeted uptake into macrophages, the primary intracellular niche of mycobacteria [222]. The conjugated nanocarriers significantly improved intracellular killing of *M smegmatis* and BCG in comparison with free rifampicin, demonstrating the utility of macromolecular conjugation and receptor‐mediated delivery for overcoming biofilm‐like aggregates within host cells.

It has been demonstrated that rifampicin‐encapsulated silk fibroin nanoparticles immobilized with antibiofilm enzymes can disrupt the *M. smegmatis* biofilm and facilitate the anti‐bacterial action of Rifampicin (RIF) [223]. The immobilized enzymes remained highly active and were able to erode *M smegmatis* biofilms, reducing their thickness by approximately 98% within 6 h. This enzymatic disruption significantly enhanced the penetration and bactericidal activity of rifampicin compared with the free drug, representing a direct enzyme‐plus‐antibiotic nanoplatform specifically validated against mycobacterial biofilms.

A nanoparticle‐based delivery system was developed using biocompatible Thymolconjugated Chitosan Zinc Ferrite Nanoparticles [224], showing enhanced biocompatibility and antibacterial activity against *M smegmatis* biofilms compared to THY alone. Also, the nanoconjugates induced Reactive Oxygen Species (ROS)‐mediated damage to the bacterial cell membrane, effectively inhibiting bacterial replication and dormancy with low cytotoxicity toward the human kidney cell line.

Composite nanoparticles encapsulating cellulase and levofloxacin use PLGA shells to deliver both an antibiotic and a biofilm‐degrading enzyme [225]. When combined with ultrasound, these NPs increase biofilm permeability, generate reactive oxygen species, and significantly reduce biofilm biomass and viability in vitro and in vivo, demonstrating a dual mechanism of physical disruption and enhanced drug penetration [226]. Similarly, silk fibroin nanoparticles immobilized with enzymes (e.g., β‐glucosidase, phytase) and loaded with rifampicin disrupt biofilm extracellular polymeric substances, reduce biofilm thickness, and enhance antibiotic action [227].

N‐acetylcysteine‐chitosan nanoparticles grafted with antimicrobial peptides and loaded with rifampicin exhibit potent inhibition of multidrug‐resistant Mtb clinical isolates, revitalizing drug efficacy by targeting both planktonic and biofilm‐associated bacteria [228]. Chitosan nanoparticles loaded with tangeretin or dihydroartemisinin disrupt cell walls and inhibit both planktonic and biofilm forms of Mtb, including resistant strains, with high biocompatibility [229]. Aptamer‐modified niosomes carrying propolis extract specifically target Mtb and inhibit growth in vitro, offering a non‐antibiotic, biofilm‐targeted approach [230].

Macrophage membrane‐coated polymeric nanoparticles encapsulating photothermal agents enable dual targeting of granulomas and Mtb. Upon near‐infrared laser irradiation, these NPs eradicate Mtb and alleviate lung pathology more effectively than standard antibiotics, representing a precise, non‐antibiotic strategy for biofilm disruption [231]. Fullertube (carbon nanomaterial) dispersions disrupt mycobacterial cell walls and inhibit biofilm formation by up to 90%, providing a novel, non‐traditional nanomaterial approach [105]. Inhalable porous silica‐based micro‐nano carriers loaded with clofazimine dissolve in lung fluid to generate nanoparticles, achieving high local drug concentrations and efficient delivery to both extracellular and intracellular Mtb, including biofilm‐associated bacteria [232]. This dual‐action system enhances mycobacterial killing and minimizes systemic toxicity.

## 5 Conclusions

Mycobacterial biofilms constitute a major barrier to effective antimicrobial therapy, not only in tuberculosis but across pathogenic mycobacterial species, where biofilm‐associated growth promotes persistence and tolerance to chemotherapy. The evidence reviewed in this work underscores that biofilm formation in mycobacteria is not a uniform process, but rather a spectrum of species‐specific strategies shaped by distinct extracellular matrix compositions, lipid remodeling pathways, and regulatory networks. In particular, marked differences between Mtb and NTM, including GPL‐dominated architectures in rapidly growing species and polysaccharide‐ and eDNA‐rich matrices in members of the MAC, highlight the limitations of extrapolating antibiofilm concepts across the genus. The therapeutic strategies discussed—antibiotic–antibiotic combinations, peptide‐based interventions, and nanotechnology‐enabled delivery systems—offer complementary approaches to counteract biofilm‐associated tolerance, but their effectiveness is inherently conditioned by biofilm composition and architecture. Interventions that enhance drug penetration, destabilize extracellular matrices, or modulate bacterial metabolic states are likely to require species‐adapted optimization rather than universal application. In this context, targeting dominant matrix components such as free mycolic acids, glycopeptidolipids, or eDNA emerges as a rational, but context‐dependent, strategy. Despite significant progress, translating antibiofilm strategies into clinically effective therapies remains challenging. Future efforts should prioritize mechanistic validation in relevant in vivo infection models, systematic evaluation across diverse mycobacterial species, and integration of biofilm biology into drug development pipelines. A refined understanding of species‐specific biofilm architectures will be essential for the rational design of therapies capable of overcoming biofilm‐associated tolerance and improving outcomes in mycobacterial infections.

## Funding

This study was supported by the São Paulo Research Foundation (FAPESP 2024/23710‐8, 2023/01664‐1, 2020/16573‐3, 2021/14603‐5). National Council for Scientific and this study was financed in part by the Coordenação de Aperfeiçoamento de Pessoal de Nível Superior—Brasil (CAPES) Finance code 001.

## Conflicts of Interest

The authors declare no conflicts of interest.

## Data Availability

Data sharing not applicable to this article as no datasets were generated or analysed during the current study.
